# Outcomes associated with comorbid atrial fibrillation and heart failure in medicare beneficiaries with acute coronary syndrome

**DOI:** 10.1186/1472-6963-14-80

**Published:** 2014-02-20

**Authors:** Shih-Yin Chen, Concetta Crivera, Michael Stokes, Luke Boulanger, Jeff Schein

**Affiliations:** 1 Evidera, 430 Bedford Street Suite 300, Lexington, MA 02420, USA; 2 Janssen Scientific Affairs, LLC, 1000 Route 202, Raritan, NJ, USA

**Keywords:** Atrial fibrillation, Coronary disease, Heart failure, Medicare

## Abstract

**Background:**

Atrial fibrillation (AF) and heart failure (HF) are both common comorbid conditions of elderly patients with acute coronary syndrome (ACS), but published data on their associated clinical and economic outcomes are limited.

**Methods:**

Our study included patients from the Medicare Current Beneficiary Survey with an incident hospitalization for ACS between 03/01/2002 and 12/31/2006. Applying population weights, we identified 795 incident ACS patients, representing more than 2.5 million Medicare beneficiaries. Of this population, 13.1% had comorbid AF, and 22.9% had HF, which were identified from Medicare claims during the 6 months prior to the first ACS event (index date) Subsequent cardiovascular (CV) hospitalizations and mortality were compared using Kaplan–Meier curves. Cox proportional hazards regressions were used to estimate the relative risk of AF and HF on CV events and mortality. Healthcare costs were summarized for the calendar year in which the incident ACS event occurred.

**Results:**

HF was associated with a 41% higher risk of mortality (HR = 1.41; 95% confidence interval [CI] 1.05–1.89). Both AF (HR = 1.46; 95% CI 1.14–1.87) and HF (HR = 1.61; 95% CI 1.26–2.06) were associated with higher risks of subsequent CV events. During the year of the incident ACS event, ACS patients with comorbid AF or HF had approximately $18,000 higher total healthcare costs than those without these comorbidities.

**Conclusion:**

Using a nationally representative sample of Medicare beneficiaries, we observed a significantly higher clinical and economic burden of patients hospitalized for ACS with comorbid AF and HF compared with those without these conditions.

## Background

Acute coronary syndrome (ACS) is one of the most common cardiovascular illnesses in the United States; in 2009, close to 1.2 million hospital discharges in the United States had an ACS diagnosis [1]. ACS results in significant morbidity and mortality [2]. Atrial fibrillation (AF), the most common sustained cardiac arrhythmia, and heart failure (HF) are frequent complications of ACS [3-5]. AF and HF share many antecedent risk factors, and approximately 40% of individuals with either of these conditions will develop the other [5]. Moreover, the development of AF appears to increase the risk of mortality from HF and vice versa [3-5].

AF prevalence increases with age. The prevalence of AF in the United States, estimated to be between 2.7 and 6.1 million in 2010, is expected to rise to between 5.6 and 12 million in 2050, as the population ages [6-8]. The incremental total healthcare cost of patients with AF, when compared with those without AF, was estimated to be $26 billion in 2008 US dollars, of which $6 billion was associated with services related to the AF diagnosis [9].

According to the American Heart Association, the prevalence of HF is also increasing, from 2.8% in 2010 to a projected 3.5% in 2030, when an additional 3 million patients will be affected [10]. This represents a 215% increase in projected direct medical costs (from $24.7 billion–$77.7 billion) and an 80% increase in projected indirect costs (from $9.7 billion–$17.4 billion) related to HF between 2010 and 2030 [10].

Past studies have examined the impact of comorbid AF and HF on the mortality of patients with ACS in the real world. In a few recent studies, the occurrence of AF among hospitalized ACS patients ranged from 4.4% to 11.8%, and was associated with mortality up to 1 year post-discharge [11-13]. The Global Registry of Acute Coronary Events (GRACE), one of the largest multinational registries for ACS, also reported the impact of HF, when developed as a complication during hospitalization, in patients with ACS [14]. Although these studies have demonstrated the adverse effects of comorbid AF and HF in ACS patients in the hospital and up to 12 months post-discharge, the associated impact on outcomes beyond 1 year has not been elucidated. Eventually, these patients transition from acute care back to the community and are managed with medical therapies. Further evaluation of long-term clinical outcomes is needed, in order to address knowledge gaps and understand the true burden of disease. Moreover, there are limited published data on economic outcomes, such as readmission, which are important from the payer’s perspective, especially Medicare, because close to half of ACS patients are older than 65 years of age [15].

The objective of this study was to evaluate outcomes in patients with ACS and comorbid AF and HF in the Medicare population over an approximately 2.5-year period.

## Methods

### Data source

We analyzed data between 2001 and 2006 from the Medicare Current Beneficiary Survey (MCBS), a panel survey sponsored by the Centers for Medicare and Medicaid Services. MCBS uses a multistage, stratified sampling design to allow findings to be representative of all Medicare beneficiaries [16]. A panel of approximately 4000 beneficiaries is recruited each year and followed for 4 years, yielding data from four panels of approximately 16,000 beneficiaries in a given year for cross-sectional analysis.

MCBS collects a wide variety of data on socioeconomic characteristics and healthcare resource utilization and costs. Survey responses regarding healthcare use are combined with Medicare Part A and Part B administrative billing claims to provide a complete picture of beneficiaries’ use of health services. For each panel, 3 years of complete healthcare utilization data are collected, including, Medicare- and non-Medicare–reimbursed healthcare utilization. The medical claims reimbursed by Medicare contain information on dates of service; International Classification of Diseases, Ninth Revision, Clinical Modification (ICD-9-CM) diagnosis and procedure codes; and costs. Data analyzed were encrypted and compliant with the Health Insurance Portability and Accountability Act; therefore, the study was exempted from institutional review board review.

### Study sample selection

ACS patients were selected from the MCBS based on inpatient medical claims with an associated diagnosis of ACS (ICD-9-CM: 410.×× [except 410.×2], 411.1×, and 411.8×) in any diagnosis fields from the Medicare Part A claims between March 1, 2002, and December 31, 2006. We chose this period after the approval of clopidogrel, which became the standard of care, to reflect the current burden with all available evidence-based medical therapies. The date of the first ACS admission was denoted as the index date. Beneficiaries had to have been in the database for at least 6 months prior to the index date to be included, but those who had any ACS events during those 6 months were excluded. Patients were then stratified into cohorts based on whether they had any Medicare claims with ICD-9-CM diagnosis codes for AF (427.31) or HF (428.xx) during the 6 months prior to the index date.

### Patient characteristics

Baseline demographic and socioeconomic characteristics, including age, gender, race, region of residence, employment status, education status, income level, marital status, and living arrangements were examined. Medicare claims during the 6 months prior to the index date were used to identify hypertension and hyperlipidemia and to measure the Charlson Comorbidity Index (CCI), a score derived by weighting 17 conditions to predict mortality [17].

### Healthcare costs

MCBS combines survey responses and Medicare claims to report total healthcare costs, including both Medicare- and non-Medicare–reimbursed services. Annual healthcare costs were summarized for the calendar year in which the incident ACS event occurred. We reported combined costs of plan-paid and beneficiary out-of-pocket payments. Total healthcare costs and costs by settings, including inpatient hospital, medical provider, short-term facility, long-term facility, outpatient hospital, prescribed medicine, home health, dental, and hospice were reported.

### Study outcomes

Mortality was ascertained from hospital discharge status (actual date available) or from the survey data (reported monthly). Cardiovascular (CV)-related rehospitalizations were identified based on Medicare Part A claims with associated diagnoses (Table 1). All outcomes were evaluated from the index date until loss of follow-up (up to 2.5 years).

**Table 1:** Diagnosis codes to identify cardiovascular-related rehospitalization

**ICD-9-CM codes**	**Description**
410.×-414.×	Ischemic heart disease
420.×-429.×	Other forms of heart disease
430.×-438.×	Cerebrovascular disease
402.9×	Unspecified hypertensive heart disease
785.0	Tachycardia unspecified
785.1	Palpitations

### Analysis

Descriptive results were reported by comparing AF vs non-AF and HF vs non-HF beneficiaries. Means and standard errors were reported for continuous variables and proportions for categorical variables. Chi-squared tests were used to detect differences for categorical variables between cohorts, and Student’s t-test was used for mean age. Since healthcare costs were skewed, we used an unadjusted generalized linear regression model with log link and gamma distribution to test for statistical differences [18]. Kaplan–Meier curves were plotted for survival probability and outcome-free survival probability for CV-related readmission, censoring at loss of follow-up. The log-rank test was used to detect unadjusted differences in Kaplan–Meier curves between study cohorts. Cox proportional hazards regressions were used to assess the risk of mortality and CV-related readmission associated with comorbid AF or HF, adjusting for patients’ age, gender, race, income level, education level, marital status, and a CCI modified by the exclusion of AF and HF. Hazard ratios (HR) and 95% confidence intervals (CI) were reported. A generalized linear regression model with log link and gamma distribution was also run on total healthcare costs adjusted for the similar covariates to assess the economic burden associated ×with AF and HF. Analyses were conducted using survey command from SAS 9.2 (Cary, NC) and STATA 11.0 (College Station, TX) incorporating population weights and clustering from multistage sampling design for robust estimates of variances.

## Results

Over a 58-month period—between March 2002 and December 2006—this study identified 795 patients with ACS, representing close to 2.5 million beneficiaries. Of these patients, 13.1% had AF, and 22.9% had HF (Table 2). There were cross-cohort differences in patient characteristics when comparing HF with non-HF beneficiaries. Compared with patients without HF, those with HF were older (78.5 years vs 75.4 years, *P* = 0.001), more likely to be institutionalized (14% vs 4%, *P* < 0.001) or to have hypertension (78% vs 65%, *P* < 0.001), and less likely to be currently employed (1.6% vs 9.9%, *P* < 0.001). Most other characteristics were similar between the AF and non-AF cohorts. Both AF and HF patients had a significantly higher CCI scores than ACS patients without these conditions.

**Table 2:** Demographic and socioeconomic characteristics of Acs patients by comorbid arial fibrillation and heart failure

	**AF cohorts**			**HF cohorts**		
	**AF**	**Non-AF**	***P *** **value**	**HF**	**Non-HF**	***P *** **value**
Number of patients	106	689		198	597	
Represented number of patients (%)	332553 (13.1%)	2209658 (86.9%)		582767 (22.9%)	1959444 (77.1%)	
Age: mean (SE)	77.87 (0.95)	75.79 (0.38)	0.071	78.48 (0.70)	75.35 (0.41)	0.001
Male (%)	45.20	49.76	0.392	42.16	51.24	0.171
Race (%):			N/A			N/A
White	85.91	86.08		82.65	87.08	
Non-white	14.09	13.76		16.77	12.92	
Missing or unknown	0.00	0.15		0.58	0.00	
Region (%):			N/A			0.215
Northeast	20.36	17.68		20.45	17.32	
Midwest	33.22	23.34		20.39	25.90	
South	36.74	43.11		40.04	42.95	
West	9.68	13.47		16.01	12.08	
Puerto Rico	0.00	2.39		3.11	1.77	
Education level (%):			0.477			0.001
Less than high school	41.29	35.53		46.00	33.39	
High school or more	57.53	63.17		51.48	65.68	
Unknown/Missing	1.18	1.31		2.52	0.92	
Marital status (%):			N/A			N/A
Married	32.36	46.31		32.68	48.00	
Widowed	48.18	37.53		51.69	35.13	
Other (separated/divorced/never married)	19.46	16.15		15.63	16.87	
Missing	0.00	0.10		0.00	0.11	
Living situation (%):			0.480			<0.001
Community dwelled	92.02	93.89		86.00	95.92	
Institutionalized	7.98	6.11		14.00	4.08	
Employment status: currently employed (%)	2.97	8.80	0.077	1.61	9.94	<0.001
Income level: less than $25,000 (%)	71.66	66.56	0.268	78.15	63.98	<0.001
Charlson Comorbidity Index: Mean (SE)	3.82 (0.27)	2.23 (0.09)	<0.05	4.45 (0.20)	1.84 (0.08)	<0.05
Distribution of Charlson Comorbidity score (%):			<0.001			<0.001
0–1	14.89	43.85		10.74	48.78	
2	16.84	20.69		10.95	22.93	
3	23.29	13.24		24.13	11.70	
4+	44.97	22.23		54.19	16.58	
Hypertension (%)	75.05	66.84	0.071	77.93	64.93	<0.001
Hyperlipidemia (%)	46.48	47.58	0.842	47.93	47.29	0.877

AF, atrial fibrillation; HF, heart failure; N/A, not applicable (statistics could not be produced when any cell size was equal to zero); SE, standard error.

The ACS patients with AF had significantly higher annual total healthcare costs than those without AF, with mean costs of $66,586 and $48,031, respectively (*P* < 0.001) (Table 3). Inpatient hospital ($35,737 vs $26,385; *P* = 0.003) and medical provider costs ($12,284 vs $8,866; *P* < 0.001) were the major cost drivers and were significantly higher for patients with AF compared with those without it. Similar results were found in the HF cohort comparisons. Compared with non-HF beneficiaries, those with HF had higher total costs ($64,548 vs $46,268; *P* < 0.001), with inpatient hospital ($32,782 vs 26,070; *P* = 0.013), and medical provider costs ($10,605 vs $8,929; *P* = 0.024) being the two major components.

**Table 3:** Annual healthcare costs of ACS patients by comorbid arial fibrillation and heart failure

	**AF cohorts**			**HF cohorts**		
	**AF**	**Non-AF**	***P *** **value**	**HF**	**Non-HF**	***P *** **value**
Annual total healthcare costs: mean (SE)	$66586 (4309)	$48031 (1500)	<0.001	$64548 (3878)	$46268 (1355)	<0.001
Inpatient hospital	$35737 (2989)	$26385 (1108)	0.003	$32782 (2667)	$26070 (1005)	0.013
Medical provider	$12284 (834)	$8866 (414)	<0.001	$10605 (673)	$8929 (432)	0.024
Short-term facility	$5159 (1316)	$2891 (294)	0.036	$5111 (903)	$2615 (344)	0.005
Long-term facility	$3335 (885)	$3100 (380)	0.823	$7018 (987)	$1974 (296)	<0.001
Outpatient hospital	$5169 (1170)	$2666 (258)	0.009	$3950 (747)	$2709 (256)	0.077
Prescribed medicine	$2704 (343)	$2531 (95)	0.623	$2556 (288)	$2553 (99)	0.993
Home health	$2005 (357)	$1165 (132)	0.011	$2061 (300)	$1041 (125)	<0.001
Dental	$92 (34)	$225 (30)	0.029	$66 (16)	$250 (34)	<0.001
Hospice	$100 (58)	$202 (57)	0.287	$398 (155)	$126 (47)	0.042

AF, atrial fibrillation; HF, heart failure; SE, standard error.

**Figure 1:**
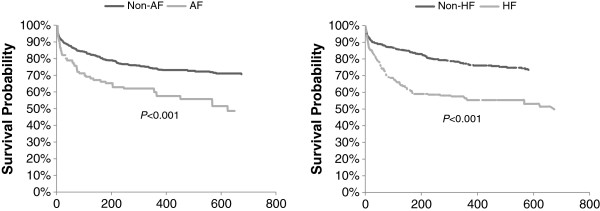
Kaplan–Meier curves for survival from admission of acute coronary syndrome patients by comorbid arial fibrillation (AF) and heart failure (HF).

**Figure 2:**
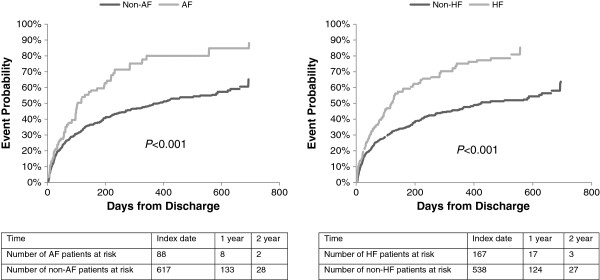
Kaplan–Meier curves for cardiovascular-readmission of acute coronary syndrome patients by comorbid arial fibrillation (AF) and heart failure (HF).

Figure 1 shows that both AF and HF patients had significantly lower probabilities of survival compared with those without these conditions (*P* < 0.001 for both). When assessing CV-related readmissions among ACS patients who were discharged alive (Figure 2), AF and HF patients also had significantly lower probabilities of outcome-free survival than their counterparts without these conditions (*P* < 0.001 for both). After adjusting for patient characteristics (Table 4), HF was associated with a 41% higher risk of mortality (HR = 1.41; 95% CI 1.05–1.89), whereas increases in mortality risk associated with AF were not significant (HR = 1.15; 95% CI 0.81–1.64). Other factors associated with a higher risk of mortality included older age (<75 years as reference; 75–84 years HR = 2.68, 95% CI 1.72–4.19; 85+ years HR = 4.98, 95% CI 3.04–8.14) and a CCI ≥4 (HR = 2.19; 95% CI 1.61–2.99 compared with CCI = 0–1). Both AF (HR = 1.46; 95% CI 1.14–1.87) and HF (HR = 1.61; 95% CI 1.26–2.06) were associated with higher risks of CV-related readmissions. No other variables in the model of CV-related readmissions were significant.

**Table 4:** Regression models for mortality and CV-readmission

	**Mortality**		**CV-related readmission**	
	**(N = 795)**		**(N = 705 among patients discharged alive)**	
	**HR**	**95% CI**	**HR**	**95% CI**
Atrial fibrillation	1.15	(0.81–1.64)	1.46	(1.14–1.87)
Heart failure	1.41	(1.05–1.89)	1.61	(1.26–2.06)
Age <75	Reference		Reference	
Age 75–84	2.68	(1.72–4.19)	0.95	(0.72–1.24)
Age 85+	4.98	(3.04–8.14)	0.99	(0.75–1.30)
Male	1.18	(0.83–1.68)	0.94	(0.71–1.24)
Female	Reference		Reference	
Race: White	Reference		Reference	
Race: Non-white	0.82	(0.54–1.25)	0.90	(0.65–1.25)
Income: ≥$25,000	0.79	(0.51–1.20)	0.99	(0.78–1.27)
Income: <$25,000	Reference		Reference	
Education: Some high school or less	Reference		Reference	
Education: High school graduate	0.79	(0.57–1.09)	0.89	(0.68–1.17)
Employment status: Currently employed	0.62	(0.25–1.56)	1.02	(0.58–1.81)
Employment status: Unemployed	Reference		Reference	
Marital status: Married	Reference		Reference	
Marital status: Widowed	1.06	(0.72–1.58)	1.05	(0.75–1.46)
Marital status: Other	1.49	(0.92–2.41)	1.24	(0.88–1.74)
CCI score = 0–1	Reference		Reference	
CCI score = 2	0.91	(0.59–1.39)	1.14	(0.84–1.54)
CCI score = 3	1.31	(0.81–2.11)	1.20	(0.85–1.70)
CCI score = 4+	2.19	(1.61–2.99)	1.29	(0.95–1.74)

HR, Hazard Ratio; CI, Confidence Interval; CCI, Charlson Comorbidity Index.

After adjusting for patient socio-demographic characteristics (Table 5), we found ACS patients with AF incurred healthcare costs that were 1.21 times of the costs in patients without AF (*P* = 0.008). ACS patients with HF incurred healthcare costs that were 1.25 times of the costs in patients without HF (*P* = 0.001). Patients with CCI score equal to or greater than 4 also had higher total healthcare costs compared to those with CCI = 0 or 1 (*P* = 0.004). Other socio-demographic variables in the model were not statistically significant.

**Table 5:** Regression model for total healthcare costs

	**Total healthcare costs**		
	**(N = 795)**		
	**Coefficient**	**Relative cost ratio**	***P *** **value**
Atrial fibrillation	0.192	1.212	0.008
Heart failure	0.227	1.255	0.001
Age <75	Reference		
Age 75–84	0.102	1.107	0.114
Age 85+	−0.023	0.977	0.761
Male	0.012	1.012	0.843
Female	Reference		
Race: White	Reference		
Race: Non-white	0.058	1.060	0.471
Income: ≥$25,000	0.106	1.112	0.183
Income: <$25,000	Reference		
Education: Some high school or less	Reference		
Education: High school graduate	−0.051	0.950	0.464
Employment status: Currently employed	0.077	1.080	0.623
Employment status: unemployed	Reference		
Marital status: Married	Reference		
Marital status: Widowed	0.029	1.029	0.664
Marital status: Other	0.148	1.160	0.083
CCI score = 0–1	Reference		
CCI score = 2	0.004	1.004	0.958
CCI score = 3	0.079	1.082	0.326
CCI score = 4+	0.269	1.309	0.004

CCI, Charlson Comorbidity Index.

## Discussion

In this nationally representative sample of Medicare beneficiaries, ACS with comorbid AF and HF was associated with greater clinical and economic burdens, compared with ACS without these conditions. The prevalence rate of AF reported in this study (13.1%) is slightly higher than in a previous study that reported prevalence rates of preexisting AF (11.4%) before hospitalization for ACS, likely because we studied an older population [11]. Similarly, the prevalence rate of preexisting HF was also higher (22.9%) than in two earlier studies, probably reflecting age differences in the study populations. However, the prevalence might not be directly comparable, as those prior studies reported HF on presentation, and one of the two studies [14] excluded patients with prior HF (prevalence rates 22.7% and 13%, respectively) [14,19]. With up to 2.5 years of follow-up, the unadjusted results in our study showed that patients with comorbid AF or HF were at a higher risk of mortality and CV-related readmission than their counterparts without AF or HF. The adjusted results, controlling for patient characteristics, showed a 46% increased risk of CV-related readmission associated with AF and a 61% risk of CV-related readmission and 41% increased risk of mortality associated with HF.

The major distinctions between our study and earlier studies examining the risk of morbidity and mortality associated with AF in an ACS population are that 1) our study sample included Medicare beneficiaries who were aged 65 years or older, and 2) we captured preexisting AF. Unlike prior observational studies with access to medical charts to determine onset of AF during hospitalization [11-13], our use of medical claims as a source of diagnosis precluded the identification of new-onset AF. Lopes *et al* and Torres *et al* demonstrated that AF complicating ACS was associated with a relative risk of 2.89 times the 6-month mortality rate and a 1.67–2.37 relative risk of mortality up to 1 year [12,13]. Although Lau *et al* distinguished between prevalent and new-onset AF, they showed an increased risk of unadjusted mortality (HR = 2.94, 95% CI 2.11–4.09) as we did; however, the investigators also found an increased risk (HR = 1.42, 95% CI 1.01–1.99) after regression adjustment, contrary to our null finding [11]. It is worth noting that their non-AF population was approximately 12 years younger (mean age = 63 years) than ours. This relatively younger age could have been responsible for the difference in results between the two studies. The additional findings related to CV-related readmission in our study would have policy implications, especially for the Medicare program, when patients survive the initial events and are transitioned back to the community. The associated risk of CV-related readmission should be considered when evaluating the cost-effectiveness of interventions or therapies in ACS patients with comorbid AF.

There is only one prior study examining the impact of HF on outcomes beyond hospitalization in patients with ACS [14]. The GRACE registry reported a higher risk of 6-month mortality in patients presenting with HF at the ACS admission, compared with those without HF (HR = 3.8; 95% CI 3.3–4.4). Our study of this nationally representative Medicare population, which followed patients for up to 2.5 years, also found a 41% higher risk of mortality associated with preexisting HF. In addition, we observed a 61% increased risk of CV-related readmission. Our findings provide further evidence of HF-associated long-term burden on elderly ACS patients in the United States and underscore the importance of treatment strategies to effectively manage and improve outcomes for these patients.

Although several earlier studies have examined costs associated with AF or HF in the Medicare population, [20-23] the incremental costs of these comorbid conditions have not been examined among ACS patients specifically. We found that ACS patients with AF or HF incurred higher costs than their counterparts without the comorbid condition. Two prior studies reported the incremental costs of AF in Medicare patients to be $14,199 and $24,235 [20,21], whereas we found the incremental cost in ACS patients to be $18,555. Differences in hospitalization rates accounted for half of the incremental cost of AF. The current literature provides very little information about incremental costs for Medicare HF patients. In this ACS population, we found the incremental cost of HF to be $18,280; two-thirds of the incremental costs can be attributed to cost differences in hospitalization and long-term care facilites. These data suggest that the economic burden associated with comorbid AF and HF in ACS patients may be related to a higher likelihood of readmission or a greater need for long-term care placement. Further assessment and interventions targeting patients with ACS and comorbid AF and/or HF may help Medicare manage the substantial economic burden.

The findings of this study should be interpreted in the context of several limitations. Healthcare costs were collected through both self-report and Medicare claims and might be underreported. Self-reported survey data are subject to recall bias. Diagnoses were determined from Medicare Part A and Part B claims, and medical services covered by other insurers or paid out-of-pocket were not captured. This might have resulted in misclassifying comorbid AF and HF. By the same token, major outcomes not captured in the dataset might lead to underestimation of the burden. While regressions were used to adjust for confounders when assessing the associated risk of comorbid AF and HF on outcomes, unobservable confounders might have led to biased estimates.

## Conclusion

Using a nationally representative sample of Medicare beneficiaries, we observed a significant clinical and economic burden among patients hospitalized for ACS who had comorbid AF and HF. Interventions and cost-effective strategies to improve outcomes and quality of care for patients with these comorbid conditions should be considered.

## Abbreviations

AF: Atrial fibrillation; HF: Heart failure; ACS: Acute coronary syndrome; CV: Cardiovascular; CI: Confidence interval; HR: Hazard ratio; MCBS: Medicare current beneficiary survey; ICD-9-CM: International classification of diseases, ninth revision, clinical modification; CCI: Charlson comorbidity index.

## Competing interests

SC, LB, MS are employees of Evidera, which provides consulting and other research services to pharmaceutical, device, government, and non-government organizations. In this salaried position, they work with a variety of companies and organizations and are precluded from receiving payment or honoraria directly from these organizations for services rendered. CC and JS are employees of Janssen Scientific Affairs, LLC (a Johnson & Johnson company), and are shareholders of Johnson & Johnson.

## Authors’ contributions

SC made signification contribution in study design, analysis and interpretation of data, and drafting and revising the manuscript. MS analyzed the data and critically reviewed the manuscript. LB participated in study design, acquisition of data, and review of the manuscript. CC and JS contributed in study conception and design, interpretation of data, and providing final approval for publication. All authors read and approved the final manuscript.

## Pre-publication history

The pre-publication history for this paper can be accessed here:

http://www.biomedcentral.com/1472-6963/14/80/prepub
